# Patterns and Factors Associated with E-Cigarette Initiation and Transition Among University Students in Al-Madinah City, Saudi Arabia: A Cross-Sectional Study

**DOI:** 10.3390/healthcare13161949

**Published:** 2025-08-09

**Authors:** Shahad Mubarak Alahmadi, Abdulmohsen Hamdan Al-Zalabani

**Affiliations:** 1 Joint Program of Preventive Medicine, Taibah University, Madinah 42353, Saudi Arabia; 2 Department of Family and Community Medicine, College of Medicine, Taibah University, Madinah 42353, Saudi Arabia; azalabani@taibahu.edu.sa

**Keywords:** electronic nicotine delivery systems, cigarette smoking, nicotine, vaping, students, tobacco products

## Abstract

**Background/Objectives**: Electronic cigarettes (e-cigarettes) have gained popularity among young adults globally, but only limited research exists on usage patterns in Saudi Arabia. This study investigated the prevalence, patterns, and factors associated with e-cigarette use initiation and transition to cigarette smoking among university students in Saudi Arabia. **Methods**: A cross-sectional survey was conducted among 537 university students in Al-Madinah city during the 2024–2025 academic year. Data were collected using a self-administered online questionnaire assessing e-cigarette use patterns, motivations, susceptibility to future cigarette smoking, and demographic characteristics. Multivariable logistic regression analysis was used to identify factors associated with e-cigarette use. **Results**: E-cigarette prevalence was substantial: 11.7% of participants were current users, 5.4% were former users, and 27.9% were ever-users, compared with 5.8% current cigarette smokers. Males had significantly higher use rates than females (20.9% vs. 4.6%; *p* < 0.001). Senior students showed the highest current use (21.1%). Among ever-users, the primary motivations were enjoyment (29.3%), the possibility of use where smoking is banned (26.1%), and social acceptability (21.7%). Most users (73.0%) used high-nicotine liquids (≥20 mg/mL), and 55.4% vaped daily. Multivariable logistic regression revealed that e-cigarette use was independently associated with male gender (adjusted OR = 4.0), higher academic year (OR = 1.24), current smoking (adjusted OR = 3.45; 95 CI: 1.54–7.82), and lower harm perception (adjusted OR = 1.69; 95 CI: 1.16–2.51). Susceptibility to future cigarette smoking was 18.9% overall but much higher among current e-cigarette smokers (59.5%; *p* < 0.001). **Conclusions**: E-cigarette use was prevalent among Saudi university students, particularly males and senior students, with patterns suggestive of nicotine dependence. Strong associations with cigarette smoking and high-concentration nicotine use raise concerns about dual use and addiction potential.

## 1. Introduction

Electronic cigarettes (e-cigarettes) have emerged as a significant public health concern, particularly among young adults (18–24 years). These rechargeable inhaler devices, filled with e-liquid—containing nicotine, flavors, propylene glycol, and vegetable glycerine—have evolved over four generations since their invention [1,2]. Since 2007, they have been globally marketed, but were not legally regulated until 2016 [3,4].

There is a lack of evidence on the long-term health effects of e-cigarettes, and it cannot be claimed that they are harm-free. E-cigarettes contain many chemical substances that are known to cause lung and cardiovascular diseases [5,6,7]. The Centers for Disease Control and Prevention (CDC) identified an outbreak of lung injury and death associated with the use of e-cigarettes, with more than 2800 cases reported by February 2020 [8]. In addition to cardiorespiratory diseases, e-cigarettes can also cause burn injuries related to explosions [9].

Young adults face unique risks from e-cigarette initiation. The developing brain remains vulnerable to nicotine until approximately age 25, with exposure potentially harming cognitive development, attention, learning, and mood regulation [10]. E-cigarette use during young adulthood is associated with an increased risk of transitioning to combustible cigarettes, with meta-analyses showing three-fold higher odds of subsequent cigarette smoking among young adult e-cigarette users [11]. Additionally, the high nicotine concentrations in modern e-cigarettes can lead to the rapid development of addiction [12]. Furthermore, young adults often underestimate addiction risk while overestimating their ability to quit, making them particularly vulnerable to long-term nicotine dependence.

In recent years, there has been a dramatic increase in the use of e-cigarettes, particularly among adolescents and young adults [13,14,15]. A systematic review and meta-analysis of global e-cigarette use among students showed the highest current rates in Europe at 12.7%, followed by North America at 12% and Asia at 11.7%, with lifetime prevalence reaching 22.0% globally [16]. The highest prevalence of use is among young adults [17,18]. Saudi Arabia’s latest Global Adult Tobacco Survey in 2019 showed an overall prevalence of ever using e-cigarettes of 3.1%, with current use at 0.8% [19]. Studies have been conducted to assess the prevalence among young adults in various regions in Saudi Arabia. A study conducted in 2020 at Jazan University, located in the southwestern region of the country, showed the prevalence of e-cigarette use among undergraduate students as 21% [20]. Another study conducted in Jeddah, a major city on the Red Sea coast, found use at 27.7% among health science college students [21].

E-cigarette use is driven by multiple factors. Marketing through social media platforms significantly influences uptake [22,23,24]. Common user-reported motivations include curiosity, social influences, perceived reduced harm compared with tobacco cigarettes, smoking cessation, and stress relief [13,25,26,27,28,29]. Several studies suggest that e-cigarettes may act as a gateway to the use of cigarettes [30,31,32]. According to one systematic review and meta-analysis, non-smoking individuals who used e-cigarettes had a three-fold higher likelihood of initiating combustible cigarette smoking compared with those who never used e-cigarettes (pooled, adjusted OR 3.19 (95% CI 2.44 to 4.16)) [33].

To understand e-cigarette use patterns, several behavioral theories provide relevant frameworks. The Theory of Planned Behavior (TPB) suggests that attitudes, subjective norms, and perceived behavioral control influence intentions and behaviors. In the context of e-cigarettes, this translates to harm perceptions (attitudes), peer influence (subjective norms), and accessibility and ability to quit (behavioral control) [34,35,36]. The Health Belief Model (HBM) suggests that perceived susceptibility, severity, benefits, and barriers shape health behaviors. For e-cigarette use, this includes perceptions of addiction risk (severity), social acceptability and enjoying e-cigarettes (benefits), and availability restrictions (barriers) [37].

Understanding the patterns and factors associated with e-cigarette initiation and subsequent transition to cigarette smoking among young adults is important for developing effective prevention strategies. While several studies have examined e-cigarette use globally, there is limited research on specific behavioral patterns in Saudi Arabia, particularly in the region of Madinah. This study, therefore, aimed to investigate the patterns and factors associated with e-cigarette initiation and transition to cigarette smoking among young adults in this region.

## 2. Materials and Methods

### 2.1. Study Design and Setting

This analytical cross-sectional study was designed to examine patterns of e-cigarette use and associated factors among young adults. This study included male and female young adults (>18 years old) enrolled in any college major listed at a governmental university in Madinah during the 2024–2025 academic year.

### 2.2. Sampling and Sample Size:

A convenience sampling technique was employed by distributing the study questionnaire through the official student email list as well as to student groups and clubs. The desired sample size was calculated using Open Source Epidemiologic Statistics (Version 3.01) for Public Health tools based on the following assumptions: a confidence interval of 95%, margin of error of 5%, and anticipated frequency of 24% based on previous cross-sectional studies of e-cigarette use among college students. The total sample size required was estimated as 280 participants [38]. To account for a 40% non-response rate typical for online surveys, the required sample size was adjusted to 280/0.6 = 466 students. We therefore aimed for 466 participants and achieved 537 responses, providing enhanced statistical power for subgroup analyses.

### 2.3. Measures

For the purpose of this study, we used the terms “electronic cigarettes,” “e-cigarettes,” and “vaping devices” interchangeably to refer to battery-powered devices that heat a liquid (e-liquid) to create an aerosol that users inhale. This definition encompasses all generations of electronic nicotine delivery systems (ENDS).

Data for this study were collected during the academic year from February to May 2025 from 537 students using an online, self-administered questionnaire. The questionnaire was accessible through Microsoft Forms and distributed via university email to all students at Taibah University. Written informed consent was obtained from all participants.

The questionnaire collected sociodemographic characteristics, including age, gender, college, academic year, and marital status.

E-cigarette use status was classified into three primary categories. Never-users: participants who reported never using e-cigarettes; former users: participants who reported past e-cigarette use but no current use; current users: participants who reported current e-cigarette use. Based on responses to the item “Have you ever, even once, used an electronic cigarette or any other vaping device?”, participants were further classified through follow-up branching questions. Those who responded that they currently use e-cigarettes (either alone or with traditional tobacco) were classified as current users, while those who reported past use but no longer use them were classified as former users. Never-users were identified based on selecting either “No tobacco use” or “Traditional tobacco only” in the branching item, indicating they were not using e-cigarettes at the time of the survey. This classification may have included participants who had never used e-cigarettes, as well as those who used traditional tobacco but had never tried e-cigarettes. For analytical purposes, ever-users refer to both current and former users combined. The difference between the number of ever-users and the combined total of current and former users was due to the survey’s branching logic and partial item non-response.

Among participants who reported ever using e-cigarettes, additional items were used to assess patterns and frequency of use. Age at initiation was assessed with the question, “At what age did you first try an e-cigarette?” Frequency and duration of use were evaluated through the items, “Do you currently use electronic cigarettes or any other vaping devices on a daily basis, less than daily, or not at all?” and “How long have you used electronic cigarettes or any other vaping devices on a daily basis?” For current users, further questions were asked about their daily habits, including: “On average, on days that you use it, how many times do you pick up your electronic nicotine product to use it, whether you take one puff or several?”, “Each time you pick up your electronic nicotine product to use it, about how many puffs do you take?”, “On the days that you use electronic nicotine products, would you say you take your first puff of the day within the first 30 min after you wake up?”, and “What strength is the e-liquid that you mainly use in your electronic cigarette or vaping device?” Participants were also asked, “When you first started using electronic nicotine products, which flavor did you use?”

Reasons for e-cigarette use—including both initiation and current use—were explored through a list of possible motivations. Options included curiosity, peer or family influence, perceived reduced harm compared with conventional cigarettes, attractive flavors, use as a smoking cessation aid, stress relief, social media influence, perceived lower nicotine content, cost advantages over conventional cigarettes, a desire to reduce conventional cigarette consumption, marketing exposure, and the ability to use e-cigarettes in places where cigarette smoking is prohibited.

Susceptibility to transition to cigarette smoking use was measured using four validated items: “Do you think that you will smoke a cigarette soon?”, “Do you think you will smoke a cigarette in the next year?”, “Do you think that in the future you might experiment with cigarettes?”, and “If one of your best friends were to offer you a cigarette, would you smoke it?”. Respondents must answer “definitely not” to all four susceptibility items to be classified as non-susceptible. Students who had any other response to any item were classified as “susceptible” [39].

Attempts to quit e-cigarette use were assessed using two questions: “How many times have you tried to quit smoking in the past 12 months?” and “Are you planning to quit smoking?” Finally, perceived harm of e-cigarettes addiction was measured by asking participants, “Do you think addiction to vaping is harmful on its own, even if it doesn’t cause physical health problems?” with response options including: “Not at all,” “Yes, a little,” “Yes, a lot,” and “Don’t know.”

The survey was developed by researchers, and items were adapted from the Global Adult Tobacco Survey and previous studies. The items related to susceptibility to smoking were adopted from Pierce et al. (1996) [39,40,41]. A pilot study was conducted with a small sample of the target population. This pilot study assessed the feasibility and evaluated the questionnaire/data collection process. 

### 2.4. Statistical Analysis

All data cleaning and statistical analyses were conducted in SPSS (Version 27, IBM Corp., Armonk, NY, USA); regression analysis was conducted using R Studio (Version 2024.09, Posit PBC, Boston, MA, USA). Continuous age data were grouped into three five-year groups (18–22, 23–27, 28+), and academic year was collapsed into three categories (first year, second to fourth years, and fifth and sixth years). Finally, the ever-use of e-cigarettes was categorized into “never” versus “former” and “current”, serving as the primary outcome. For missing data, we have used a complete case analysis approach.

Descriptive analyses summarized the distribution of each variable, both overall and stratified by smoking status or susceptibility group. Frequency tables displayed counts and percentages within each variable category. Differences across groups were tested using Pearson’s chi-squared test, while Fisher’s exact test was used whenever expected cell counts fell below five. Statistical significance was assessed at the 0.05 level with two-tailed *p*-values.

For the multivariable analysis, demographic factors, smoking-pattern measures, motivation items, and device-type indicators were initially entered into a multivariable logistic regression model examining associations with ever-use of e-cigarettes. To ensure stable estimation, any potential associated factor with more than 30% missing data or that collapsed to a single category among its observed cases was dropped before modeling. We then performed backward stepwise selection based on Akaike’s Information Criterion (AIC), sequentially removing the factor whose removal most improved the AIC until no further reduction was possible. At each step, the AIC was recorded to illustrate the trade-off between model complexity and goodness of fit. Model fit was assessed using the likelihood ratio test, Tjur’s R^2^, and Hosmer–Lemeshow goodness-of-fit tests.

### 2.5. Ethical Considerations

This study was approved by the Institutional Review Board of the College of Medicine at Taibah University (approval number IRB00010413). Electronic informed consent was obtained from all participants, who were fully informed about this study’s purpose and their rights, including voluntary participation and withdrawal without penalty. To ensure anonymity and confidentiality, no personal identifiable information was collected, and IP addresses were not recorded. Data confidentiality was ensured through secure data storage and anonymous data handling. All research procedures adhered to the Declaration of Helsinki on ethical principles for medical research involving human subjects [42].

## 3. Results

The 537 students in this survey were mainly unmarried young adults (96.3%), and more than half were female (56.4%). Most (76%) were between 18 and 22 years old, and almost two-thirds (65%) studied outside the health sciences. Nearly two-thirds were in their second to fourth academic year, while the rest were split between first- and fifth- or sixth-year students.

E-cigarette prevalence was substantial, with 11.7% being current users, 5.4% being former users, and 27.9% being ever-users. Cigarette smoking was reported by 3.7%, while 9.7% used e-cigarettes exclusively. The dual use of e-cigarettes and cigarettes was uncommon (2%). Risk perception was high, with 90.1% rating e-cigarette addiction as “very harmful” and 6.7% as “harmful.” (Table 1).

Among ever-users, the most common reasons were enjoyment (29.3%), the ability to use where smoking is banned (26.1%), and greater social acceptability than smoking (21.7%). Notably, pleasure-seeking motivations predominated over health-related reasons, with smoking cessation cited by only 16% of users.

Refillable liquid tanks were the most common type of device (14.1%), followed by disposable e-cigarettes (9.8%), with cartridge and modular systems being rare (2.2% and 3.3%, respectively).

Usage patterns among current users suggested established habits. Nearly half took their first puff within 30 min of waking, the majority used high-nicotine products (≥20 mg/mL), and 55.4% used e-cigarettes daily. Long-term use was common, with 48.9% having used e-cigarettes for over two years. Quit intentions were limited among current users (*n* = 63): only 33.3% planned to quit within six months and 12.7% after six months, while 30.2% had no plans to quit or were unsure. (Table 2).

E-cigarette use showed clear demographic differences. Males were over four times more likely to be current users than females, and prevalence increased significantly with academic progression, peaking among senior students (Table 3). Current cigarette smokers have higher e-cigarette use than non-smokers (35.5% vs. 10.3%; *p* < 0.001). Risk perception was also associated: only 9.5% of students rating e-cigarette addiction “very harmful” were current users, compared with 36.1% of those rating it “harmful” (*p* < 0.001). Students with uncertain or low-risk perceptions also showed elevated use rates.

Among current users, initiation was most often attributed to curiosity (46%) and peer influence (44.4%). For the initial flavor choice, mixed fruit predominated (74.6%), followed by tobacco (17.5%) and mint or candy flavors (14.3% each). Other flavors were rarely chosen. (Table 3).

After excluding current and former cigarette smokers, 18.9% (*n* = 92) of students showed susceptibility to future cigarette smoking. Susceptibility varied dramatically by e-cigarette use status (*p* < 0.001) with a four-fold increase from never to current users (14.2% among never-users, 45.0% among former users, and 59.5% among current e-cigarette users). No significant differences were found across other variables (Table 4).

The multivariable logistic regression model (*n* = 537; Tjur’s R^2^ = 0.143) identified several factors independently associated with ever-use of e-cigarettes (current or former use versus never). First, gender emerged as a strong associated factor, as female students had 75% lower odds of e-cigarette use compared with males (OR = 0.25, 95% CI 0.14–0.42, *p* < 0.001). Faculty type did not significantly influence uptake (OR = 0.93, 95% CI 0.56–1.58, *p* = 0.787), nor did age in years (OR = 1.03 per additional year, 95% CI 0.92–1.12, *p* = 0.575). In contrast, each advancing academic year was associated with a 24% increase in the odds of e-cigarette use (OR = 1.24, 95% CI 1.04–1.50, *p* = 0.022), suggesting that senior students were more likely to experiment with or adopt e-cigarettes.

Current cigarette smoking was also a strong associated factor, as cigarette smokers had more than three times the odds of e-cigarette use compared with non-smokers (OR = 3.45, 95% CI 1.54–7.82, *p* = 0.003). Finally, risk perception played a significant role: we found that for each one-point decrease in perceived harmfulness of e-cigarette addiction, the odds of e-cigarette use rose by 69% (OR = 1.69, 95% CI 1.16–2.51, *p* = 0.007). Together, these findings indicate that male gender, higher academic seniority, concurrent cigarette smoking, and lower perceived risk of addiction independently increased the likelihood of e-cigarette uptake among university students. (Table 5).

## 4. Discussions

This study found substantial e-cigarette use among Saudi university students (11.7% current users, 27.9% ever-users), with marked gender disparities and strong associations with cigarette smoking. These findings contribute important regional data to the global understanding of e-cigarette adoption patterns, particularly in a cultural context where tobacco use has significant social and religious constraints [43,44].

### 4.1. Prevalence of Electronic Cigarette Use

Our prevalence rates fall within the range reported across Saudi universities but are lower than some previous estimates. Current use rates vary considerably: 12.2% at Alfaisal University in a sample of medical students, (mean age 20) with 39% males and 61% females [45], 20.1% at Shaqra University (mean age 20.5) with 66.2% males and 33.8% females [46], and 24% in an earlier Taibah study [38]. A recent meta-analysis found wide variation (7.2–79.8%) in Saudi studies, with our university-based sample aligning with the conservative end of this range [47]. Qatar University reported a 14% prevalence (mean age 23.48) with 37.2% males and 62.8% females [48]. Generally, our results confirm trends observed across the kingdom, such as male predominance, an overlap with conventional smoking, and a mix of motives relating to pleasure, convenience, and smoking cessation.

In 2025, Alaboodi et al. pooled 15 studies conducted in Saudi Arabia with a total of 6736 participants. They found the prevalence of e-cigarette use to be 36.6%, with a very wide range from 7.2% in university students to 79.8% among young adults. Our 11.7% prevalence is near the lower bound and is consistent with the reported university student subset (7.2%). The reasons for this wide variation could include methodological heterogeneity, as many earlier studies sampled vape-shop patrons or online followers, which likely inflated the upper range. Our lower prevalence, derived from a university student population, therefore provides a more conservative estimate of campus e-cigarette use [47].

Our prevalence rates are similar to global estimates from a recent meta-analysis that reported 22.0% lifetime and 10.2% current use [16].

### 4.2. Perception of Harm and Susceptibility

Most students (90.1%) rated e-cigarette addiction as ‘very harmful,’ representing a substantial increase from a previous study, in which 71% of respondents considered e-cigarettes safe [33]. This high-risk perception may reflect recent intensified public health campaigns. Notably, perceived harm is strongly associated with use; with each one-point reduction in harm perception, the odds of e-cigarette use increased by 69% (OR = 1.69, *p* = 0.007). However, despite high-risk awareness, users showed behaviors associated with dependence, including daily use and early-morning use of e-cigarettes, suggesting that harm perception alone may not prevent sustained use once initiated.

Through the Health Belief Model lens, our results show a disconnect between perceived severity (90.1% rating addiction as ‘very harmful’) and actual behavior. This suggests that perceived benefits (enjoyment and social acceptance) and reduced barriers (concealment and availability) may outweigh perceived risks, particularly among males and senior students.

The high susceptibility among current e-cigarette users compared with never-users suggests that e-cigarette use is associated with increased openness to future cigarette smoking, supporting concerns about e-cigarettes serving as a gateway to cigarette smoking use, These findings align with Khouja et al.’s meta-analysis showing that young non-smokers who had tried e-cigarettes were significantly more likely to initiate cigarette smoking [49].

### 4.3. Reasons and Patterns of E-Cigarette Use

In our sample, enjoyment was the primary motive for e-cigarette use (29.3%), followed by the ability to use e-cigarettes in places where smoking is banned (26.1%) and greater social acceptability compared with cigarettes (21.7%). Only 16% cited smoking cessation, in contrast with an earlier Taibah study in which cessation was the most common motive (49%) [38]. This difference may reflect variations in user demographics or study populations. Studies of specific subgroups show different patterns; male smokers in Al-Ahsa prioritized peer influence (56%) and stress relief (39%) [50], while Alfaisal medical students frequently cited cessation (29%) [45].

Peer influence was minimal in our sample (1.1%). This contrasts with Qatar, where having a smoking friend increased the odds of e-cigarette use more than seven-fold [48]. Meanwhile, Jordanian e-cigarette users most often cited smoking cessation (26.5%), curiosity (22%), and perceived lower harm (20.5%) [51]. These differences may reflect variation in cultural attitudes toward tobacco use and harm reduction messaging across the region.

Nearly half of the e-cigarette users surveyed use e-cigarettes within 30 min of waking, 55.4% use an e-cigarette daily, and nearly half had sustained use for ≥2 years. These patterns suggest moderate-to-high nicotine dependence, consistent with reports from international studies. Ramo et al. noted that use was most common in males, those who had recently attempted to quit, and those with stronger nicotine dependence (e.g., smoking within 30 min of waking) or high motivation to quit [52]. In a Polish study by Jankowski et al., they found that young adults who used e-cigarettes showed significantly greater nicotine dependence than those who smoked, with mean Fagerstrom Test for Nicotine Dependence (FTND) scores of 3.5 for exclusive e-cigarette users versus 1.6 for cigarette smokers [53].

In our study, dual use was widespread, as 35.5% of current cigarette smokers also used e-cigarettes, whereas only 10.3% of non-smokers used e-cigarettes; cigarette smoking increased the odds of e-cigarette use by more than three-fold. Both studies indicated that among highly educated young adults, e-cigarettes not only foster dependence equal to or greater than that of cigarette smoking but also frequently co-occur with smoking, thereby amplifying total nicotine exposure and complicating cessation efforts. Long-term cessation intent was limited in our sample, as only 7.9% of current e-cigarette users indicated an intention to quit within the next month, and 14.2% within six months. A substantial majority, 83%, reported either no plans to quit or uncertainty over the issue.

### 4.4. Participant Characteristics

Sex differences were pronounced in our data; 20.9% of males versus 4.6% of females currently used e-cigarettes. Our multivariable model confirmed this disparity, as being female reduced the odds of ever using e-cigarettes by 75%. Salari et al. estimate that globally, 4.9% of boys and 1.6% of girls are current e-cigarette users, a male-to-female ratio of roughly 3:1 [54]. This compares to a 4.5:1 male-to-female ratio in our sample, indicating particularly strong gender effects in this Saudi population.

This gender disparity appears to be consistent across developing countries. Malaysian studies found 95% of e-cigarette users were male [55], while Chinese studies reported male odds ratios of 3.51 in Shanghai [56], and a proportion of 58.3% male users in Guangzhou [45]. However, some exceptions exist: Qatar University found minimal gender differences (16.2% vs. 12.8%) [48], suggesting that cultural restrictions on female nicotine use may vary regionally.

E-cigarette use increased with academic progression, from 5.4% among first-year students to 21.1% in fifth and sixth years, paralleling the age-related increase from 9.6% at ages 18–22 to 20% in those aged 23+. This pattern suggests that older, more senior students have higher sustained use rates, possibly reflecting greater autonomy and established behavioral patterns.

Our findings align with the Theory of Planned Behavior. The strong gender differences (males 20.9% vs. females 4.6%) likely reflect different subjective norms in Saudi culture. The primary motivations of enjoyment (29.3%) and social acceptability (21.7%) represent positive attitudes toward e-cigarettes, while the ability to use where smoking is banned (26.1%) demonstrates how perceived behavioral control facilitates use.

Based on these theoretical insights, interventions should target changing attitudes by emphasizing immediate negative consequences, including addiction; addressing subjective norms through peer-led campaigns, particularly for male students; and increasing perceived barriers through stronger enforcement of vaping restrictions on campus.

The 100% excise tax implemented in 2017 [57], which doubled e-cigarette prices, likely influenced our findings. The tax may have deterred price-sensitive experimenters, potentially explaining our moderate prevalence rates. The predominance of high-nicotine products (73% using ≥ 20 mg/mL) may reflect users maximizing nicotine per purchase. Notably, only 9.8% cited cost advantages as a motivation for use.

This cross-sectional study has some limitations. It was carried out at a single public university; therefore, the findings may not be generalizable to private universities or young adults outside of higher education. The use of convenience sampling may have introduced selection bias, potentially limiting the generalizability of our findings to other university populations in Saudi Arabia. The cross-sectional design precludes causal inferences about relationships between variables. Moreover, nicotine dependence was assessed through behavioral proxy measures rather than validated scales such as the FTND. While our indicators (morning use, daily frequency, and nicotine concentration) align with established dependence markers, the absence of validated scales limits definitive conclusions about addiction severity. Finally, data were collected shortly after a national excise tax rise on electronic nicotine products, and prevalence may shift as the market adjusts. Despite these limitations, several features represent strengths of the work. The questionnaire was distributed university-wide and achieved good participation (537 students), providing a substantial sample size. A comprehensive set of items captured frequency, motives, risk perception, susceptibility to future smoking, and behavioral signs of dependence, allowing multifactor analysis rarely undertaken in Saudi Arabia. Data were gathered after recent fiscal and regulatory actions, offering an up-to-date snapshot. In addition, systematic comparison with regional and international reports placed the findings in a wider epidemiological context.

## 5. Conclusions

In this study of undergraduates, one in nine students was an active e-cigarette user, and more than one in four had tried the product at least once. E-cigarette use was more common in males, cigarette smokers, and senior students, and often persisted without a clear plan to stop. While most participants recognized the potential harm, use behaviors were suggestive of some nicotine dependence. These results underline the need for continuous surveillance, focused education, and integrated cessation support to address e-cigarette use among Saudi university students.

## Figures and Tables

**Table 1:** Overall distribution of the students’ characteristics and smoking prevalence.

Characteristic	Category	Frequency (%)
1. Demographics:		
Sex	Male	234 (43.6%)
	Female	303 (56.4%)
Academic field	Health sciences colleges	187 (34.8%)
	Non-health sciences colleges	350 (65.2%)
Marital status	Single	515 (95.9%)
	Married	20 (3.7%)
	Divorced	2 (0.4%)
Age group	18–22	408 (76.0%)
	23–27	114 (21.2%)
	≥28	15 (2.8%)
Academic year	First year	112 (20.9%)
	2nd–4th year	335 (62.4%)
	5th–6th year	90 (16.8%)
2. Smoking prevalence:		
E-cigarette use status	Never	445 (82.9%)
	Former e-cigarette use	29 (5.4%)
	Current e-cigarette use	63 (11.7%)
Current cigarette smoking status	Yes	31 (5.8%)
	No	506 (94.2%)
Current tobacco/e-cig description	No tobacco use	431 (80.3%)
	Both cigarettes and e-cigarettes/vapes	11 (2.0%)
	E-cigs/vapes only	52 (9.7%)
	Quit e-cigs/vapes	23 (4.3%)
	Cigarettes only and ex-vape	6 (1.1%)
	Cigarettes only	14 (2.6%)
Ever Used E-Cigarette	Yes	150 (27.9%)
	No	387 (72.1%)
3. Perceived harm of e-cigarettes		
Perceived harm of e-cig addiction	Very harmful	484 (90.1%)
	Harmful	36 (6.7%)
	Don’t know	12 (2.2%)
	Not harmful at all	5 (0.9%)

**Table 2:** Distribution of e-cigarette use patterns, reasons for use, and quit attempts among e-cigarette users (current or former).

Characteristic	Category	Frequency (%)
Reasons for e-cigarette use	To quit smoking tobacco	15 (16.3%)
	I enjoy e-cigarette smoking	27 (29.3%)
	To avoid going back to smoking tobacco	3 (3.3%)
	More socially acceptable	20 (21.7%)
	Less expensive	9 (9.8%)
	Less harmful to people around me	13 (14.1%)
	I can use it where tobacco smoking is not allowed	24 (26.1%)
	Less harmful than smoking tobacco	13 (14.1%)
	It comes in flavors I like	14 (15.2%)
	It smells good	8 (8.7%)
	To reduce the number of tobacco cigarettes I smoke	8 (8.7%)
	A friend or family member uses them	1 (1.1%)
	Other reasons	1 (1.1%)
What main type of E-cigarette do you use	Refillable liquid tank	13 (14.1%)
	Disposable device	9 (9.8%)
	Replaceable cartridges	2 (2.2%)
	Modular system	3 (3.3%)
First puff within 30 min	Differ by day	21 (33.9%)
	No	12 (19.4%)
	Yes	29 (46.8%)
E-cigs contain nicotine	No	1 (1.6%)
	Yes	61 (98.4%)
Nicotine concentration	6 mg (0.6%) or less	4 (6.3%)
	From 7 mg (0.7%) to 11 mg (1.1%)	2 (3.2%)
	From 12 mg (1.2%) to 19 mg (1.9%)	4 (6.3%)
	20 mg (2.0%)	8 (12.7%)
	More than 20 mg (2.0%)	38 (60.3%)
	I don’t know	7 (11.1%)
E-cig use frequency	<daily	17 (18.5%)
	Daily	51 (55.4%)
	Not at all	7 (7.6%)
	Quit	17 (18.5%)
E-cig use duration	1–2 yr	24 (26.1%)
	1–3 months	8 (8.7%)
	4–11 months	9 (9.8%)
	<1 month	6 (6.5%)
	>2 yr	45 (48.9%)
Last e-cig use	1–2 yr	5 (5.4%)
	1–3 months	5 (5.4%)
	4–12 months	6 (6.5%)
	<1 month	11 (12.0%)
	>2 yr	10 (10.9%)
	Still using	55 (59.8%)
Plan to quit using e-cigarettes (Among current e-cigarette users only; *n* = 63)	Yes, in the coming month	12 (19%)
	Yes, in 1–6 months	9 (14.3%)
	Yes, after 6 months	8 (12.7%)
	I don’t know	11 (17.5%)
	No plans for quitting	8 (12.7%)
	Not answered the question	15 (23.8%)
Reasons for initiating e-cigarette use (Among current e-cigarette users only; *n* = 63)	I was curious about them	29 (46%)
	A friend was/still is using them	28 (44.4%)
	To get the effect of nicotine	20 (31.7%)
	Because they cost less than other tobacco products like regular cigarettes	7 (11.1%)
	To try to quit using other tobacco products like regular cigarettes	10 (15.9%)
	I was/still am feeling anxious, stressed, or depressed	12 (19%)
	Because they are available in flavors such as mint, fruit, candy, or chocolate	5 (7.9%)
	Because I can use them without anyone noticing at home or school	15 (23.8%)
	A family member was/still is using them	6 (9.5%)
	Because they are easier to obtain than other tobacco products like regular cigarettes	3 (4.8%)
When you first started using e-cigarettes/vapes, what flavor did you use? (Among current e-cigarette users only; *n* = 63)	Mixed fruit	47 (74.6%)
	Mints	9 (14.3%)
	Grapes	1 (1.6%)
	Tobacco	11 (17.5%)
	Candy	9 (14.3%)
	Coffee or other drinks (soda and power drinks)	2 (2.3%)
	Vanilla	1 (1.6%)
	Others	1 (1.6%)
	I don’t know	1 (1.6%)

**Table 3:** Students’ characteristics stratified by status of e-cigarette use.

Characteristic	Category	Never	Former	Current	*p*-Value
Gender	Male	166 (70.9%)	19 (8.1%)	49 (20.9%)	<0.001 *
	Female	279 (92.1%)	10 (3.3%)	14 (4.6%)	
Academic field	Health sciences colleges	156 (83.4%)	8 (4.3%)	23 (12.3%)	0.684
	Non-health sciences colleges	289 (82.6%)	21 (6.0%)	40 (11.4%)	
Marital status	Single	427 (82.9%)	26 (5.0%)	62 (12.0%)	0.259
	Married	16 (80.0%)	3 (15.0%)	1 (5.0%)	
	Divorced	2 (100.0%)	0 (0.0%)	0 (0.0%)	
Age group	18–22	349 (85.5%)	20 (4.9%)	39 (9.6%)	0.058
	23–27	85 (74.6%)	8 (7.0%)	21 (18.4%)	
	≥28	11 (73.3%)	1 (6.7%)	3 (20.0%)	
Academic year	First year	102 (91.1%)	4 (3.6%)	6 (5.4%)	0.008 *
	2nd–4th year	278 (83.0%)	19 (5.7%)	38 (11.3%)	
	5th–6th year	65 (72.2%)	6 (6.7%)	19 (21.1%)	
Current cigarette smoking Status	Yes	14 (45.2%)	6 (19.4%)	11 (35.5%)	<0.001 *
	No	431 (85.2%)	23 (4.5%)	52 (10.3%)	
Perceived harm of e-cig addiction	Very harmful	415 (85.7%)	23 (4.8%)	46 (9.5%)	<0.001 *
	Harmful	20 (55.6%)	3 (8.3%)	13 (36.1%)	
	Don’t know	6 (50.0%)	3 (25.0%)	3 (25.0%)	
	Not harmful at all	4 (80.0%)	0 (0.0%)	1 (20.0%)	

* *p*-value < 0.05 indicates statistically significant difference.

**Table 4:** Susceptibility to cigarette smoking among students who never smoked cigarettes.

Variable	Category	Not Susceptible	Susceptible	*p*-Value
Sample	*n* (%)	396 (81.2%)	92 (18.9%)	
E-cigarette use	Never	370 (85.9%)	61 (14.2%)	<0.001
	Former e-cigarette use	11 (55.0%)	9 (45.0%)	
	Current e-cigarette use	15 (40.5%)	22 (59.5%)	
Gender	Male	155 (80.3%)	38 (19.7%)	0.702
	Female	241 (81.7%)	54 (18.3%)	
Age	18–22	314 (83.1%)	64 (16.9%)	0.107
	23–27	73 (73.7%)	26 (26.3%)	
	≥28	9 (81.8%)	2 (18.2%)	
Academic year	First year	93 (86.1%)	15 (13.9%)	0.208
	2nd–4th year	250 (80.7%)	60 (19.4%)	
	5th–6th year	53 (75.7%)	17 (24.3%)	
Academic field	Health sciences colleges	144 (82.3%)	31 (17.7%)	0.631
	Non-health sciences colleges	252 (80.5%)	61 (19.5%)	
Marital status	Single	380 (80.9%)	90 (19.2%)	0.392
	Married/Divorced	16 (88.9%)	2 (11.1%)	

**Table 5:** Multivariable logistic regression of factors associated with e-cigarette use among university students in Al-Madinah city.

	Current E-Cigarette Use		
Associated Factors	Adjusted Odds Ratios	95% CI	*p*
Gender			
Males	Ref.	-	-
Females	0.25	0.14–0.42	<0.001
Academic Field			
Health sciences	Ref.	-	-
Non-health sciences	0.93	0.56–1.58	0.787
Academic year			
Lower academic year	Ref.	-	-
Higher academic year	1.24	1.04–1.50	0.022
Current cigarette smoking			
No	Ref.	-	-
Yes	3.45	1.54–7.82	0.003
Age (years) (per year increase)	1.03	0.92–1.12	0.575
Believing e-cig addiction is harmful (per unit increase)	0.59	0.40–0.86	0.007
Observations	537		
likelihood ratio test	*p* < 0.001		
Hosmer–Lemeshow	*p* = 0.205		
R^2^ Tjur	*p* = 0.143		

## Data Availability

Data supporting the findings of this study are available from the corresponding author upon reasonable request.
